# The Effect of Dentine Desensitizing Agents on the Retention of Cemented Fixed Dental Prostheses: A Systematic Review

**DOI:** 10.3390/medicina59030515

**Published:** 2023-03-06

**Authors:** Mohammed E. Sayed

**Affiliations:** Department of Prosthetic Dental Sciences, College of Dentistry, Jazan University, Jazan 45142, Saudi Arabia; drsayed203@gmail.com or mesayed@jazanu.edu.sa; Tel.: +966-506529134

**Keywords:** dentin hypersensitivity, desensitizing agent, retention, luting cements, bond strength, GLUMA, glass ionomer cement, resin cement, tooth preparation

## Abstract

*Background and Objectives:* The use of desensitizing agents (DA) after tooth preparation to prevent hypersensitivity is well documented in the literature. A fixed dental prosthesis (FDP) should have good retention to be successful. Inadequate retention may result in microleakage, secondary caries, and, eventually, dislodgement of the FDP. The effect of DAs on the retention of FDPs has been widely studied in the literature, but the results are conflicting. Thus, this study aimed to conduct a systematic review to assess the effect of dentine desensitizing agents, used to prevent post-cementation hypersensitivity, on the retention of cemented FDPs. The null hypothesis framed was that there is no effect of dentine desensitizing agents on the retention of cemented FDPs. The focused PICO question was as follows: “Does the application of dentine desensitizing agents (I) affect the retention (O) of cemented fixed dental prosthesis (P) when compared to non-dentine desensitizing groups (C)”? *Materials and Methods:* Four electronic databases were systematically searched and, on the basis of the predefined inclusion and exclusion criteria, 23 articles were included in this systematic review. A modified CONSORT scale for in vitro studies was used to assess the quality of the selected studies, as all included studies were in vitro studies. *Results:* Most of the studies compared the effect of more than one type of DA on retention. The results of the selected studies varied due to differences in the composition of tested dentine DAs and types of luting cements. *Conclusions:* Within the limitations of this study, it can be concluded that the retention values of FDPs cemented using zinc phosphate cement were reduced with most of the DAs, whereas retention values increased when GIC, resin-modified GIC, and resin cements were used with the majority of DAs. These findings are important, as they can guide dentists in selecting the DA before cementing the crowns with the luting agent of their choice, without compromising the retention of the crowns.

## 1. Introduction

A fixed dental prosthesis (FDP) is a common treatment modality for replacing missing teeth and for transforming unhealthy teeth into functional and esthetically pleasing ones [1]. To prepare a tooth for an FDP, the coronal tooth structure is prepared, which involves the removal of 1–2 mm of the tooth structure [1]. This procedure leads to the opening of millions of dentinal tubules [2,3,4]. Preparation also reduces the thickness of the dentine (depending upon the type of preparation and location of preparation), which increases the permeability of the dentine [3,4,5,6,7]. This causes pulpal irritation and post-operative hypersensitivity [7,8].

Heat generation [9,10,11], desiccation [9,10,11], aggressive tooth preparation [9], microleakage underneath provisional restoration [11,12], and the acidic pH of many luting agents [10,11,13] lead to irritation of the dentinal tubules, which in turn irritate the pulp and cause discomfort to the patient in the form of sensitivity.

The use of desensitizing agents (DA) after tooth preparation to prevent hypersensitivity has been well documented in the literature [14,15,16,17]. Various generations of DAs have been used in the past, and they have shown promising results in reducing post-preparation sensitivity [14,15,16,17,18,19,20]. These include 2-hydroxyethyl-methacrylate (HEMA), urethane dimethacrylate (UDMA), Tolnyl ethyl glycidal dimethacrylate (TEGMA), N-Olyglycine glycidyl methacrylate (NTG-GMA), biphenyl dimethacrylate (BPDM), 5% glutaraldehyde + HEMA, Low and highly filled resins, etc. [14,15,16,17,18,19,20]. Recent studies have demonstrated that new types of DAs have comparable desensitizing effects on dentine. These include nano-hydroxyapatite (n-HAp) [21,22,23], photobiomodulation therapy (PBM) with a low-level infrared laser [24], nano-sized carbonate apatite (n-CAP) [25], zinc-containing desensitizer [26], etc. Most of the DAs block the opening of the bulk of the dentinal tubules and make the dentinal surface smooth by filling the irregularities, thereby decreasing the sensitivity [14,15,16,17].

For an FDP to be successful, it should have good retention. Multiple factors affect the retention of FDP, including adequacy of tooth preparation, impression-making, fit and precision of the retainer, space and type of luting agent [27,28,29,30,31]. Inadequate retention may result in microleakage, secondary caries, and dissolution of luting agent [30,31,32,33]. A dislodged FDP is considered to be a failure from the patient’s perspective, and he/she may doubt the reliability of the treatment provided by the dentist.

The effect of DAs on retention of FDPs has been widely studied in the literature, but the results are conflicting. Studies by Johnson et al. [34], Jalandar et al. [18], Chandavarkar et al. [8] and Himashilpa et al. [35] have reported higher retention values when GIC was used with Gluma DA, whereas lower retention values were reported by Swift et al. [36], Yim et al. [37] and Sipahi et al. [38]. Similarly, studies by Chandavarkar et al. [8] and Pilo et al. [39] reported higher retention values when GIC was used with pro-argenine-based DAs, whereas Himashilpa et al. [29] reported lower retention values for the same combination. Retention of FDP was reported to be affected by the combined effect of the type of luting agent and DA.

To the best of our knowledge, this is the first systematic review to assess the effect of DAs on the retention of cemented FDPs. The findings are important, as they can guide dentists in selecting the DA before cementing the FDPs with the luting agent of their choice, without compromising retention. Thus, the objective of this study is to conduct a systematic review to assess the effect of dentine desensitizing agents, used to prevent post-cementation hypersensitivity, on the retention of cemented FDPs. The null hypothesis framed is that there is no effect of dentine desensitizing agents on the retention of cemented FDPs.

## 2. Materials and Methods

### 2.1. Permission and Registration

For the planning of this systematic review, registration in the International Prospective Register of Systematic Reviews (PROSPERO) was applied for (CRD388403). The preferred reporting items for systematic reviews and meta-analyses (PRISMA) guidelines were used to structure this systematic review [40].

### 2.2. Search Criteria

Studies were selected based on the following inclusion and exclusion criteria. All published in vitro and in vivo studies in the English language that compared the effect of dentine desensitizers on the retention of full- and partial-coverage FDPs after cementation were included in this systematic review. Studies that were under trial, unpublished abstracts, commentaries, letters to editors, case reports, or dissertations were excluded. Exclusion criteria also included studies in languages other than English, animal studies, studies comparing the sensitivity or bond strength of luting agents to dentine after the application of dentine desensitizers, and studies evaluating materials under trial.

The focused PICO question was as follows: “Does the application of dentine desensitizing agents (I) affect the retention (O) of cemented fixed dental prosthesis (P) when compared to non-dentine desensitizing groups (C)”?

P: Cemented fixed dental prosthesis

I: Dentine desensitizer application

C: Non-dentine desensitizer application

O: Retention of crowns

Four electronic databases (MEDLINE/PubMed, Scopus, Cochrane Library, and Web of Science–Core Collection) were systematically searched in October 2022 for relevant titles with respect to the formulated PICO question. Details of the keywords and Boolean operators used in the search strategy are listed in Supplementary Table S1. On the basis of the requirements of each electronic database, slight amendments were made to the search strategy. A reference list of articles was searched manually for further relevant titles.

### 2.3. Screening, Selection of Studies, and Data Extraction

After performing the search on the selected electronic databases, the collected titles and their abstracts were independently examined by two reviewers (MES and MM). Duplicate titles were removed, and the titles and abstracts of the remaining studies were assessed against the preset inclusion and exclusion criteria. Full texts of the selected titles were reviewed and the studies that met the inclusion criteria were collected. Two reviewers (MES and MM) discussed the selected studies, and any disputed studies were discussed with third reviewer (S.J.) to resolve disagreements. The reference list of the selected studies was searched manually to check for any supplementary relevant studies that met the requirements. Relevant data were extracted from the studies that fulfilled the inclusion criteria and were tabulated in a self-designed table. Table 1 is a self-designed master table containing information related to Author, Year and Country; Study Design; Sample Size; Abutment Type; Specimen Fabrication Technique; Type of Framework (Single Crown/3 Unit FPD); Crown/FPD Fabrication Technique; Control; Intervention; Name of DA (Manufacturer); Main Chemical Composition of DA; Type of Cement, Trade Name and Manufacturer; Test and Machine Used; Mean Tbs/Retentive Strength; Primary Outcomes; Secondary Outcomes; and Authors’ Suggestions/Conclusions/Inferences.

### 2.4. Quality Assessment of the Included Studies

A modified CONSORT scale for in vitro studies [50,51] was used to assess the quality of the selected studies. The standards of different sections of the published studies can be assessed using the checklist, which includes 14 items. The items included were as follows: “Item 1: Abstract containing structured summary of study design, methodology, results, and conclusions; Item 2a: Introduction should have scientific background and detailed explanation of rationale; Item 2b: Introduction should have study objectives with a defined hypothesis; Item 3: Methodology should contain approach used in the experiment with sufficient details to enable replication; Item 4: Precisely stated primary and secondary outcomes to enable comparison; Item 5: Details of how sample size was determined; Item 6: Details of how random allocation sequence was generated; Item 7: Method used for random allocation concealment; Item 8: Who implemented randomization? Item 9: If randomization is performed, how was blinding followed? Item 10: Statistical assessment; Item 11: Results outcome and estimation; Item 12: Study limitations; Item 13: Details related to funding; Item 14: Details related to the availability of study protocol, if available” (Table 2).

## 3. Results

### 3.1. Identification and Screening

**Figure 1:**
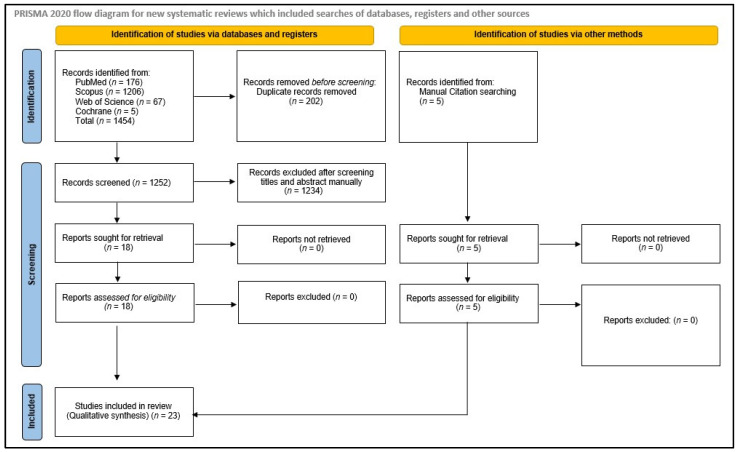
PRISMA flow-chart demonstrating the article selection strategy, preferred reporting items for systematic reviews and meta-analyses.

An electronic search in PubMed, Scopus, Cochrane, and Web of Sciences resulted in 1454 hits. Of these, 202 articles were duplicates and, hence, were removed. After screening the titles and abstracts of these articles, 1234 articles were removed. The full texts of the remaining 18 articles were reviewed by two authors and, after discussion, all 18 articles were selected for final inclusion in the study. Five articles were added after manual search of the references of the selected articles. Thus, finally, a total of 23 articles were included that satisfied all the selection criteria and addressed the PICO question (Figure 1).

### 3.2. Characteristics of the Selected Studies

A total of 23 in vitro studies were assessed via a selection process in this systematic review. Out of the 23 total studies, 10 studies were conducted in India, 4 in the USA, 2 in Israel, and 1 each in Georgia, Iran, Saudi Arabia, Syria, Turkey, Germany, and Switzerland. The most recent studies were published in 2022, and the oldest was published in 1996 (Table 1). All 23 studies demonstrated comparative analysis of the test and control groups and assessed the effect of desensitizing agents on the retention of cemented crowns. The sample size in the selected studies ranged from *n* = 20 [31] to *n* = 420 [35].

Twelve out of the twenty-three studies used human molars, whereas eleven studies used human premolars for evaluating the bond strength of the cemented crowns/copings. In most of the studies, the taper for preparation of the tooth was kept between 6° and 20°. All studies used full-coverage crowns/copings for retention assessment. The materials used to fabricate these full-coverage retainers were base metal alloys in seventeen studies [8,11,12,18,20,31,34,35,37,38,39,43,44,45,46,47,48], noble/high noble alloys in three studies [36,43,45], and zirconia ceramic in three studies [10,19,49]. In most of the studies involving metal alloys, the fabrication technique of crowns/copings was lost wax casting, whereas in two studies, an additive manufacturing technique (3D printing) was used [39,47]. In all of the studies using zirconia crowns/copings, the subtractive manufacturing technique (CAD/CAM milling) was used for fabrication [10,19,49]. (Table 1).

The majority of the studies compared the effect of liquid-based DAs on retention, whereas three studies compared the effect of lasers as DA along with liquid-based DAs [8,38,44]. Most of the studies compared the effect of more than one type of DA on retention. Nearly thirteen studies used glutaraldehyde-based DAs [8,11,12,18,19,31,34,35,36,45,46,48,49], six used arginine-based DAs [8,10,35,39,46], five used CPP-ACP-based DAs [8,18,35,43,47], and three studies each used phosphoric acid-based [20,36,42] and resin-based [11,12,37] DAs. Few studies assessed the effect of other types of DAs (D-TMR-based, HEMA NTG-GMA-based, etc.) on the retention of cemented crowns. (Table 1).

Most of the studies compared the bond strength using different types of luting cement [10,11,12,18,19,20,34,35,36,37,39,41,42,43,44,47,48,49]. Commonly used cements include zinc phosphate, glass ionomer cement, resin-modified GIC, and resin cement. Only one study also compared polycarboxylate cement along with the above-mentioned cements [41]. (Table 1).

### 3.3. Findings of Quality Analysis

As all of the studies selected in this systematic review were in vitro studies, the modified CONSORT scale [50,51] for in vitro studies was used to perform quality analysis of the selected studies, on the basis of which 61.7% (213/345) of the entries were positively rated (Table 2). Entries related to the quality of the abstract (Item 1), the introduction (Item 2a, 2b), the intervention (Item 3), the outcomes (Item 4), the statistical methods used (Item 10) in the methodology section, and the results section (Item 11) were rated positively for all of the selected articles. Thirteen studies reported their limitations (Item 12), eight reported details related to the sources of funding (Item 13), six briefly reported details on the randomization method (Item 6), only two reported of the method used for sample size calculation (Item 5), and one study made the full study protocol accessible (Item 14). One study reported steps taken to conceal the random allocation (Item 7), but none of the studies reported having taken steps necessary to prevent bias, such as who made the random distribution sequence (Item 7) and how blinding was performed (Item 9). Overall, the quality of the selected articles was good, with a moderate risk of bias.

### 3.4. Results of Individual Studies

The results of the selected studies varied due to differences in the composition of the tested dentine DAs and the types of luting cements. After the application of liquid-based DAs, the studies reported an increase in the retention of crowns when cemented with resin cements [19,31,36,37,41,46,49], when cemented with GIC [8,18,20,39,42,43], and when cemented with RMGIC [18,37,45,47,48]. However, the use of DAs with ZPC was reported in almost all of the studies to decrease the retention of cemented crowns [11,18,20,37,39,41,42,43]. The studies also reported a reduction in retention when GIC or resin cements were used with specific DAs [35,37,41,46,47,48]. The use of a laser as a DA was reported to reduce the retention of crowns when cemented using GIC [8,38,44]. However, Kumar et al. [44] reported that retention increased when laser was used as a DA and resin cement was used for the cementation of retainers (Table 1).

## 4. Discussion

Tooth preparation for full-coverage FDP involves reduction of the coronal tooth structure. Hypersensitivity is commonly reported after cementation of crowns/FPDs on prepared vital teeth [52]. Dentine desensitizing agents are commonly applied on the teeth before cementation to prevent this hypersensitivity, but their effect on the retention of cemented crowns is still debatable [8,10,11,12,18,19,20,31,34,35,36,37,38,39,41,42,43,44,45,46,47,48,49]. The current systematic review is the first of its kind to evaluate the quality of the published literature assessing the effect of DAs on the bond strength of cemented crowns. All 23 included articles were in vitro prospective randomized controlled trials [8,10,11,12,18,19,20,31,34,35,36,37,38,39,41,42,43,44,45,46,47,48,49]. The findings of the 23 included studies suggest that the use of DAs affects the bond strength of cemented crowns, and that the results vary according to the type of DA and the cement used for cementation, thereby rejecting the proposed null hypothesis.

Multiple reasons for post-cementation hypersensitivity have been postulated in the literature, including the opening of dentinal tubules, the chemical composition and the initial low pH of the luting cements, microleakage and bacterial leakage due to polymerization shrinkage of luting agents, desiccation of the tooth, hydraulic pressure on tubules during luting, higher permeability due to smear layer removal, etc. [47,53,54,55,56]. To minimize this post-cementation hypersensitivity, DAs are commonly used before cementation. These DAs can be in the form of liquids or lasers [8,12,38,42,43,44,45,46,47]. They act in multiple ways, which include blocking the opening of dentinal tubules, reducing inflammation, depolarization of the nerves, etc. [11,47]. The protective layer formed by DA can affect the retention of cemented crowns by reducing the micromechanical retention tags [15,16,44].

When evaluating the retention of crowns cemented with ZPC, most studies report a decrease in retention values after the application of DAs. [11,18,20,35,37,39,41,42,43]. ZPC uses irregularities on the dentine surface to attain mechanical retention. Application of most of the DAs blocked these irregularities, thus making the surface smooth and causing a decrease in retention. Meanwhile, in three studies, the retention values were slightly higher [11,18,34]. All three studies used the GLUMA desensitizer, which has been reported to obliterate the bulk of dentinal tubules and infiltrate into them as plugs [57]. This does not alter the irregularities on the dentine and, thus, does not reduce the retention of cemented crowns [18,57].

With GIC as a luting agent, studies have reported contrasting results for retention values with the application of DAs. The type of DA used affected the retention values to a great extent. The retention values were reported to be higher in all studies that used GC Tooth Mousse [8,18,35,43] and One Step [20,36,42] as a DA before cementation. The mechanism of bonding of GIC is chemico-mechanical. The use of GC Tooth Mousse makes the dentine surface smooth, thus helping to increase retention values, as GIC bonds better on smoother surfaces [18,35]. Higher retention values with the application of One Step DA may be due to the chemical affinity of GIC towards HEMA monomers of resin DAs. Thus, after the interface of GIC and resin has been set, it is reported to be like that of RMGIC [20,36,42]. Four studies reported higher retention values when Gluma DA was used [8,18,34,35], whereas three studies reported lower retention values [36,37,38]. The increase in retention values was proposed to be due to the chemical affinity of GIC towards resin sealers containing glutaraldehyde and HEMA [18], whereas the reduction in retention values was proposed to be due to GLUMA being a non-polymerizing resin-based sealer that fills the irregularities of dentine, thus preventing the formation of chelating bonds with dentine [37]. The use of Colgate Sensitive Pro-Relief was reported in two studies to increase retention values [8,39] and in one study to reduce retention values [35]. Chelation between polyalkenoic chains in GIC and calcium carbonate in Pro-Arging-based DAs was presumed to be a possible cause of higher retention values [39], whereas interference in bonding due to the delicate plugs formed by the DAs was presumed to be the cause of poor retention values [35]. Systemp DA increased the retention values in one study [35] and reduced them in the other [48]. The binding of calcium and fluoride minerals released from GIC with the system protein plugs was proposed to be the cause of higher retention values [35]. All Bond [37,41] and lasers [8,38,44] reduced retention values in all of the studies that used them as DAs. Lasers were reported to cause desiccation of the collagen fibrils, as well as producing micro-explosions on the top surface of the dentinal tubules, leading to smear layer formation. These changes interfere with the chemical bonding of GIC with dentine, thus reducing the retention values [44,58].

Retention values when RMGIC is used as a luting agent after DA application varied in different studies. In general, the use of Systemp DA increased retention values [35,48]. Gluma as DA increased retention values in three studies [18,36,45] and decreased them in two studies [35,37]. The use of Tooth Mousse [18,43] or Colgate Sensitive Pro-Relief [10,35] as DAs had no effect on retention values. The binding of protein plugs formed by Systemp with resin tags was proposed to be the cause of higher retention values when Systemp DA was used with RMGIC [35,48]. The increase in retention values with Gluma was proposed to be due to the chemical affinity of RMGIC towards resin sealers containing HEMA [18,45].

Most of the studies reported higher retention values for crowns cemented using resin cements after the application of different DAs [19,31,34,35,37,41,46,49]. Polymerization between the HEMA complex (at the dentine–DA junction) and resin cement [31,59,60], the rewetting properties of HEMA, the buffering capacity of resins [61], and micro-mechanical bonding between protein plugs formed by DAs and resin tags [35,62] may be possible reasons for increased retention values when RC is used with DAs. The use of Pro-Argenine [10] and lasers [44] as DAs was reported to cause no change in retention values when RC was used. It has been proposed that lasers increase the calcium ions on the surface of the dentine, which may increase chelating reactions and resin cements, partially decalcifying the smear layer (formed after laser treatment), thus forming resin tags [44,63].

In the absence of DAs, the retention values were reported to be highest for RC, followed by RMGIC and GIC, while ZPC displayed lowest retention values [18,20,34,35,37,39,41,42,43,44,47,48]. Adhesive bonding between calcium ions and monomers in resin cement was shown to possess increased retention values compared to other cements [64,65].

The type of dentine desensitizing agent used in the selected studies influenced the outcome of this systematic review. With time, new generations of DAs have evolved that have better handling and properties. The comprehensive search and selection protocol is a key feature of this systematic review. Limitations of this systematic review include a moderate to high risk of bias in the selected studies, the wide variety of tested materials, and the differences in testing conditions. The current systematic review aimed to discuss the effects of DAs on the retention of crowns. The effect of these DAs on hypersensitivity also needs to be addressed, as this is an important parameter when selecting the best DA for patients before crown cementation to minimize post-operative sensitivity.

## 5. Conclusions

The following conclusions can be drawn on the basis of this systematic review:

1. The type of dentine desensitizing agent and luting agent used affect the retention values of the cemented FDPs.
2. In general, the retention values of FDPs cemented using zinc phosphate cement are reduced with most of the DAs, whereas retention values increase when GIC, resin-modified GIC, and resin cements are used with the majority of DAs.
3. Blinding protocols should be followed in future in vitro studies to avoid bias.
4. Dentists should have knowledge regarding the compatibility of DAs and luting cements in order to provide the best treatment to their patients.

## Figures and Tables

**Table 1:** General characteristics and specific results of the included studies.

Author, Year and Country	Study Design	Sample Size	Abutment Type	Specimen Fabrication Technique	Type of FDP (Single Crown, 3 Unit FPD) and Fabrication Technique	Control	Intervention	Name of DA (Manufacturer)	Main Chemical Composition	Type of Cement, Trade Name and Manufacturer	Test and Machine Used	Mean TBS (N)/ Retentive Strength (MPa)	Primary Outcomes	Secondary Outcomes	Authors’ Suggestions/Conclusions/ Inferences
Mausner et al., 1996, USA [41]	In vitro	*n* = 96 (16 per group)	Human Third molars	Finish line: rounded shoulder/bevel Axial height: 5 mm Taper: 6–10° Spacer: 3 coats Ageing: No	Full metal silver–palladium alloy copings (Ceradela 2, Metalor, Neuchatel, Switzerland) Fabrication technique: lost wax casting	No DA applied	Application of DA before final cementation	(A) Imperva bonding agent (IBA) (Shofu Dental Corp., Menlo Park, CA, USA) (B) All-Bond (AB) desensitizing agent (Bisco Inc., Itasca, IL, USA)	(A) HEMA & UDMA & TEGMA (B) NTG-GMA & BPDM	(i) ZPC (Flecks Mizzy, Mizzy, Inc., Cherry Hill, NJ, USA) (ii) PCC (Duralon, Espe-Premier, Norristown, PA, USA), (iii) GIC (Ketec Cem Maxicaps, Espe-Premier, St. Paul, MN, USA), (iv) RC (NM)	Retention values, UTM	Retention values (N) (A) ZPC: 383.28 ± 62.17 (B) ZPC + IBA DA: 354.89 ± 84.06 (C) ZPC + AB DA: 187.48 ± 50.18 (D) PCC: 335.97 ± 54.29 (E) PCC + IBA DA: 388.26 ± 34.53 (F) PCC + AB DA: 42.85 ± 14.24 (G) GIC: 234.74 ± 64.70 (H) GIC+ IBA DA: 135.73 ± 41.39 (I) GIC + AB DA: 211.37 ± 39.43 (J) RC: 289.25 ± 116.10 (K) RC + IBA DA: 485.05 ± 117.21 (L) RC + AB DA: 406.06 ± 132.61	Retention values: RC + IBA DA > RC + AB DA > PCC + IBA DA > ZPC > ZPC + IBA DA > PCC > RC > GIC > GIC + AB DA > ZPC + AB DA > GIC+ IBA DA > PCC + AB DA	Retention values: ZPC > PCC > RC > GIC	In general, application of DA reduced the retention in most of the tested specimens when cemented with ZPC, PCC or GIC, whereas retention increased when RC was used.
Swift et al., 1997, USA [36]	In vitro	*n* = 30 (10 per group)	Human molars	Finish line: NM Axial height: 4 mm Taper: 2.4° per wall Spacer: NM Ageing: No	Full metal silver–palladium copings (Ney-Oro 76, Ney Dental International) Fabrication technique: lost wax casting	No DA applied	Application of DA before final cementation	(A) One step (Bisco Dental Products, Schaumburg, IL, USA) (B) Gluma (Heraeus Kulzer, South Bend, IN, USA)	(A) phosphoric acid with Benzalkonium Chloride (B) glutaraldehyde and HEMA	(i) ZPC (Hy-Bond, Shofu Inc., Koyoto, Japan) (ii) GIC ((Fuji I, GC America Inc., Alsip, IL, USA) (iii) RMGIC (Vitremer Luting Cement, 3M Dental Products Division, St. Paul, MN, USA)	Mean force for removing crown, UTM	Mean force for removing crown (N) (A) ZPC: 587 ± 400 (B) ZPC + One step DA: 479 ± 215 (C) ZPC + Gluma DA: 449 ± 277 (D) GIC: 788 ± 401 (E) GIC + One Step DA: 872 ± 342 (F) GIC + Gluma DA: 653 ± 234 (G) RMGIC: 685 ± 156 (H) RMGIC + One Step DA: 713 ± 191 (I) RMGIC + Gluma DA: 748 ± 306	Mean force for removing crown GIC + One Step DA > GIC > RMGIC + RMGIC + Gluma DA + One Step DA > RMGIC > GIC + Gluma DA > ZPC > ZPC + One step DA > ZPC + Gluma DA	Retention GIC > RMGIC > ZPC	Use of DA does not affect the retentive properties of the three tested luting cements.
Johnson et al., 1998, USA [34]	In vitro	*n* = 60 (10 per group)	Human molars	Finish line: chamfer Axial height: 4 mm Taper: 20° Spacer: 3 coats Ageing: No	Full base metal alloy copings (Olympia porcelain metal alloy, Jelenko Dental Products, Armonk, NY, USA) Fabrication technique: lost wax casting	No DA applied	Application of DA before final cementation	Gluma Desensitizer sealer (Heraeus/Kulzer, Dental Products Division, South Bend, IN, USA)	5% glutaraldehyde + HEMA	(i) ZPC (Fleck’s, Mizzy Inc., Cherry Hill, NJ, USA) (ii) GIC (Ketac-Cem Maxicap, ESPE Gmbh, Seefeld, Germany) (iii) Modified RC (Resinomer, Bisco, Inc., Schaumburg, IL, USA)	Failure stress, UTM	Failure stress (MPa) (A) ZPC: 6.3 (B) ZPC + Gluma DA: 6.4 (C) GIC:9.1 (D) GIC + Gluma DA:10.1 (E) Modified RC: 12.1 (F) Modified RC + Gluma DA: 12.6	Failure stress: RC + DA > RC > GIC + DA > GIC > ZPC + DA > ZPC	RC > GIC > ZPC	Application of Gluma DA for desensitizing treatment does not affect retention of crowns cemented with the tested luting agents.
Yim et al., 2000, Georgia [37]	In vitro	*n* = 144 (12 per group)	Human molars	Finish line: Chamfer Axial height: 4 mm Taper: 26° Spacer: 2 coats Ageing: No	Full metal Ni-Cr crown Fabrication technique: lost wax casting	No DA	Application of DA before final cementation	(A) PD (All-Bond 2, BISCO Dental Products, Schaumburg, IL, USA) (B) NPD (Gluma Desensitizer, Heraeus Kulzer, South Bend, IN, USA)	(A) Photopolymerizable, resin-based DA (B) Nonpolymerizing, protein-precipitating, resin-based DA	(i) ZPC (Fleck’s Cement, Mizzy Inc., Cherry Hill, NJ, USA) (ii) GIC (Ketac Cem, ESPE GmbH, Seefeld, Germany) (iii) RMGIC (Fuji Plus, GC Corporation, Tokyo, Japan) (iv) RC (Panavia 21, J. Morita, Tustin, CA, USA)	Debond Stress; UTM	Debond Stress (MPa): (A) ZPC + PD DA: 0.67 ± 0.14 (B) ZPC + NPD DA: 0.81 ± 0.11 C) ZPC: 1.68 ± 0.08 (D) GIC + PD DA: 2.23 ± 0.20 (E) GIC + NPD DA: 1.98 ± 0.23 F) GIC: 2.36 ± 0.20 (G) RMGIC + PD DA: 3.46 ± 0.26 (H) RMGIC + NPD DA: 2.81 ± 0.15 (I) RMGIC: 2.96 ± 0.18 (J) RC + PD DA: 5.68 ± 0.70 (K) RC + NPD DA: 4.12 ± 0.37 (L) RC: 4.67 ± 0.48	Debond Stress RC + PD DA > RC > RC + NPD DA > RMGIC + PD DA > RMGIC > RMGIC + NPD DA > GIC + PD DA > GIC > GIC + NPD DA > ZPC > ZPC + NPD DA > ZPC + PD DA	Debond Stress RC > RMGIC > GIC > ZPC	Application of NPD DA significantly decreased the retention strength when RC, GIC and ZPC were used. Application of PD DA significantly increased retention strength when RC and RMGIC was used. DA when used with ZPC significantly decreased retention strength.
Wolfart et al., 2003, Germany [12]	In vitro	*n* = 80 (10 per group)	Human premolars	Finish line: Chamfer Axial height: 4 mm Taper: 11° Spacer: yes Ageing: 3 days and 150 days (37,500 cycles)	Full metal nickel chromium alloy (Wiron 99, Bego, Germany) copings Fabrication technique: lost wax casting	Calcium Hydroxide DA applied	Application of DA before final cementation	(A) Gluma (Heraeus Kulzer) (B) Prompt L-Pop (3M-Espe, Seefeld, Germany) (C) Optibond FL (Kerr, Orange County, CA, USA) (D) Calcium hydroxide suspension (Merck, Darmstadt, Germany)	(A) 5% Glutaraldehyde and HEMA (B) Low filled resin sealer (C) Highly filled resin sealer	GIC (Ketac-Cem Maxicup,3M-Espe, Seefield, Germany)	Failure Stress, UTM	Failure Stress (MPa) After 3 days aging: ^##^ (A) GIC + Calcium hydroxide:6.92 (B) GIC + Gluma: 6.20 (C) GIC + Prompt L-Pop: 6.62 (D) GIC + Optibond: 4.91 After 150 days aging: ^##^ (A) GIC + Calcium hydroxide: 6.02 (B) GIC + Gluma: 5.60 (C) GIC + Prompt L-Pop: 6.9 (D) GIC + Optibond:5.01	Failure stress After 3 days ageing: GIC + Calcium hydroxide > GIC + Prompt L-Pop > GIC + Gluma > GIC + Optibond After 150 days ageing: GIC + Prompt L-Pop > GIC + Calcium hydroxide > GIC + Gluma > GIC + Optibond	-	Gluma and Prompt L-Pop DA does not affect the retention of crowns cemented with GIC when compared to calcium hydroxide application.
Johnson et al., 2004; USA [42]	In vitro	*n* = 55 (11 per group)	Human molars	Finish line:—NA Axial height: 4 mm Taper: 20° Spacer: 1 layer Ageing: 2500 cycles	Full ceramometal high noble alloy (Olympia) copings Fabrication technique: lost wax casting	No DA applied	Application of DA before final cementation	(A) One step (Bisco Dental Products, Schaumburg, IL, USA)	Phosphoric acid with Benzalkonium Chloride	(A) ZPC (Fleck’s, Keystone Industries GmbH, Singen, Germany), (B) GIC (Ketac-Cem, ESPE Gmbh, Seefeld, Germany) (C) Modified-RC (Resinomer, Schaum-burg, IL, USA)	Dislodgment stresses, UTM	Mean dislodgment stress (MPa) (A) ZPC: 3.7 ±1.0 (B) ZPC + One step DA:2.2 ± 0.8 (C) GIC: 2.7 ± 1.2 (D) GIC + One step DA: 4.2 ± 0.9 (E) Modified-RC: 6.4 ± 1.7	Mean dislodgment stress Modified RC > GIC + One step > ZPC > GIC > ZPC + One step	dislodgment stress: Modified RC > ZPC > GIC	Resin sealers reduced retention when used with ZPC and increased retention when used with GIC.
Sipahi et al., 2007, Turkey [38]	In vitro	*n* = 50 (10 per group)	Human molars	-	Full metal base metal alloy copings Fabrication technique: lost wax casting	No DA applied	Application of DA before final cementation	(A) Laser group (LAS), (B) sodium fluoride group (C) Oxagel oxalate group (D) Gluma primer group	-	GIC	TS, UTM	TS (N) (A) GIC: 261 (B) GIC + Laser DA: 223 (C) GIC + sodium fluoride DA: 208 (D) GIC + Oxagel DA: 147 (E) GIC + Gluma DA: 161	Ts: GIC > GIC + Laser > GIC + sodium fluoride > GIC + Gluma > GIC + Oxagel	-	Lee negative effect of laser treatment on retention for crowns cemented with GIC, as compared to other DA.
Jalandar et al., 2012, India [18]	In vitro	*n* = 90 (10 per group)	Human molars	Finish line: Chamfer Axial height: 4 mm Taper: 6° Spacer: 35–40 µ Ageing: No	Full metal Ni-Cr crown Fabrication technique: lost wax casting	No DA	Application of DA before final cementation	(A) GC Tooth Mousse (GC International, Itabashiku, Tokyo, Japan) (B) GLUMA desensitizer (Heraeus Kulzer, Hanau, Germany).	(A) CPP-ACP-based (B) GLU-based	(i) ZPC (Harvard cement Quick setting, Harvard Dental International GmbH, Hoppegarten, Germany) (ii) GIC (GC Fuji 1Tokyo, Japan) (iii) RMGIC (RelyX^TM^ Luting, 3M ESPE, St. Paul, MN, USA)	TBS; UTM	TBS (kg) (A) ZPC + TM DA: 25.27 ± 4.60 (B) ZPC + GLUMA DA: 27.92 ± 3.20 (C) ZPC:27.69 ± 3.39 (D) GIC + TM DA: 40.32 ± 3.89 (E) GIC + GLUMA DA: 41.14 ± 2.42 (F) GIC: 39.09 ± 2.80 (G) RMGIC + TM DA: 48.34 ± 2.94 (H) RMGIC + GLUMA DA: 49.02 ± 3.32 (I) RMGIC: 48.61 ± 3.54	TBS: RMGIC + GLUMA DA > RMGIC > RMGIC + TM DA > GIC + GLUMA DA > GIC + TM DA > GIC > ZPC + GLUMA DA > ZPC > ZPC + TM DA	TBS: RMGIC > GIC > ZPC	GLUMA DA improves retention of cast crowns with ZPC, GIC, RMGIC. Tooth Mousse DA improves retention of cast crowns with GIC, RMGIC and reduces retention for ZPC.
Stawarczyk et al., 2012, Switzerland [19]	In vitro	*n* = 144 (12 per group)	Human molars	Finish line: Shoulder Axial height: 3 mm Taper: 10° Spacer: 35–40 µ Ageing: half specimens were aged—chewing machine, 6000 cycles	Zirconia crowns Fabrication technique: CAD/CAM milled	No DA	Application of DA before final cementation	Gluma Desensitizer (Haereus Kulzer, Hanau, Germany)	HEMA, glutaraldehyde	(i) Panavia 21 (Kuraray Dental Co. Ltd., Osaka, Japan) (ii) RelyX Unicem (3M ESPE, Seefeld, Germany) (iii) G-Cem (GC, Leuven, Belgium)	TS; UTM	Tensile strength (MPa) Initial (A) Panavia 21 + Gluma DA: 2.6 ± 1.4 (B) Panavia 21: 14.1 ± 3.5 (C) RelyX Unicem + Gluma DA: 13.1 ± 2.9 (D) RelyX Unicem: 12.8 ± 2.9 (E) G-Cem + Gluma DA: 13.7 ± 4.2 (F) G-Cem: 10.7 ± 2.9 After Ageing (A) Panavia 21 + Gluma DA: 0.9 ± 0.6 (B) Panavia 21: 7.3 ± 1.7 (C) RelyX Unicem + Gluma DA: 12.8 ± 4.3 (D) RelyX Unicem: 9.1 ± 3 (E) G-Cem + Gluma DA: 13.4 ± 6.2 (F) G-Cem: 8.6 ± 2.2	Tensile strength Initial: Panavia 21 > G-Cem + Gluma DA > RelyX Unicem + Gluma DA > RelyX Unicem > G-Cem > Panavia 21 + Gluma DA After Ageing G-Cem + Gluma DA > RelyX Unicem + Gluma DA > RelyX Unicem > G-Cem > Panavia 21 > Panavia 21 + Gluma DA	TS: Panavia 21 > RelyX Unicem > G-Cem	RelyX Unicem & G-Cem (self-adhesive Resins) when used with Gluma DA displayed better long-term stability.
Patel et al., 2013, India [20]	In vitro	*n* = 55 (11 per group)	Human molars	Finish line: Chamfer Axial height: 4 mm Taper: 20° Spacer: 3 layer Ageing: 2500 cycles	base metal porcelain metal alloy (Wirobond 280, BEGO, Fabrication technique: lost wax casting	No DA applied	Application of DA before final cementation	One-Step—Resinomer, (Bisco)	phosphoric acid with Benzalkonium Chloride	(A) ZPC: (Harvard; Harvard Dental International GmbH, Hoppegarten, Germany) (B) GIC: (Vivaglass; Ivoclar vivadent Inc.,Buffalo, NY, USA) (C) Modified RC (Resinomer, Bisco Inc., Schaum-burg, IL, USA)	Removal stress, UTM	Removal stress (MPa) (A) ZPC: 3.5682 ± 0.2135 (B) ZPC + DA: 1.9209 ± 0.152 (C) GIC: 2.4082 ± 0.2581 (D) GIC + DA: 4.2609 ± 0.1963 (E) Modified RC: 6.9591 ± 0.5883	Removal stress: Modified RC > GIC + DA > GIC > ZPC > ZPC + DA	Removal stress: RC > GIC > ZPC	DA reduces retention with ZPC and increases retention with GIC.
Chandrasekaran et al., 2014, India [43]	In vitro	*n* = 81 (9 per group)	Human maxillary first premolars	Finish line: Chamfer Axial height: 4 mm Taper: 6–10° Spacer: NM Ageing: No	Full metal Ni-Cr crown Fabrication technique: lost wax casting	No DA	(A) & (B) Application of DA before final cementation	(A) Seal and protect (dentsply) (B) Tooth Mousse (GC)	(A) D-TMR & PENTA (B) CPP-ACP	(i) ZPC (Harvard cement, Harvard Dental International GmbH, Hoppegarten, Germany) (ii) GIC (GC Fuji 1, Tokyo, Japan) (iii) RMGIC (GC Fuji Plus, GC Corporation, Tokyo, Japan)	Bond strength; UTM	Mean Bond strength (MPa) (A) ZPC + SP DA: 249.25 ± 65.65 (B) ZPC + TM DA: 219 ± 49.30 (C) ZPC:295.12 ± 31.16 (D) GIC + SP DA: 345.49 ± 109.86 (E) GIC + TM DA: 421.46 ± 96.52 (F) GIC: 416.21 ± 113.10 (G) RMGIC + SP DA: 379.26 ± 114.59 (H) RMGIC + TM DA: 528.5 ± 67.65 (I) RMGIC: 537.2 ± 73.83	Mean Bond strength: RMGIC > RMGIC + TM DA > GIC + TM DA > GIC > RMGIC + SP DA > GIC + SP DA > ZPC > ZPC + SP DA > ZPC + TM DA	Mean Bond strength: RMGIC > GIC > ZPC	Retentive strength: RMGIC: Control > TM > SP GIC: TM > Control > SP ZPC: Control > SP > TM TM & SP Can be used before crown cementation using GIC or RMGIC, but not with ZPC.
Kumar et al., 2015, India [44]	In vitro	*n* = 48 (12 per group)	Human maxillary first premolars	NM	Full metal Ni-Cr crown Fabrication technique: lost wax casting	No DA	laser treatment Er, Cr: YSGG laser at 0.5 W potency for 15 s	Desensitising Laser: Er, Cr: YSGG laser (NM)	NA	(i) GIC (ii) self-adhesive RC	TBS; UTM	TBS (N): GIC: 170 ± 7.519 GIC + DA:119.08 ± 5.350 RC: 244.33 ± 11.865 RC + DA: 269.16 ± 5.184	TBS: RC + DA > RC > GIC > GIC_DA	TBS: RC > GIC	The luting agent of choice for laser DA treated dentine: self-adhesive RC.
Chandavarkar et al., 2015 India [8]	In vitro	*n* = 50 (10 per group)	human premolars	Finish line: Chamfer Axial height: 4 mm Taper: 20° Spacer: 25 µ Ageing: No	Full metal Ni-Cr crown Fabrication technique: lost wax casting	No DA	(A), (B), (D): Application of DA before final cementation (C) laser treatment Er, Cr: YSGG laser at 0.5 W potency for 45 s	(A) Gluma Desensitizer, (Haereus Kulzer, Hanau, Germany) (B) GC Tooth Mousse, Recaldent Tooth Mousse, GC Corporation, Tokyo, Ja-pan)). (C) Waterlase MD Turbo, Biolase Inc, Foothill Ranch, CA, USA) (D) Colgate Sensitive Pro-Relief in-office polishing paste, New York, NY, USA)	(A) GLU-based (B) CPP-ACP-based (C) Er, Cr: YSGG laser (D) Pro-Argin	GIC	Tensile stress; UTM	Tensile stress (MPa); (A) GLU DA + GIC: 3.87 (B) CPP-ACP DA + GIC: 4.01 (C) Laser DA + GIC:3.37 (D) Pro-Argin DA + GIC: 4.10 (E) GIC: 3.65	Tensile stress: Pro-Argin DA + GIC > CPP-ACP DA + GIC > GLU DA + GIC > GIC > Laser DA + GIC	-	Pro-Argin and CPP-ACP-based DA can be used safely without compromising the retention of cast crowns cemented with GIC. Laser as DA reduces the tensile stress when used with GIC.
Janapala et al., 2015, India [45]	In vitro	*n* = 40 (10 per group)	Human maxillary first premolars	Finish line: NM Axial height: 4 mm Taper: 20° Spacer: NM Ageing: No	Full metal nickel chromium alloy copings (Bellabond, BEGO) Fabrication technique: lost wax casting	No DA applied	Application of DA before final cementation	(A) Cavity varnish (Namuvar, Deepti Dental Products, Maharashtra, India) (B), Glutaraldehyde (Gluma-Heraeus Kulzer, Hanau, Germany), (C) Resin (AdheSE bond, Ivoclar Vivadent, Buffalo, NY, USA)	(A) Dissolved solids (B) 5% Glutaraldehyde & HEMA (C) HEMA, dimethacrylate, silicon dioxide	RMGIC (FujiCEM, GC Corporation, Tokyo, Japan)	TS, UTM	Tensile strength (N) (A) RMGIC: 2.627 ± 1.1887 (B) RMGIC + Varnish: 1.968 ± 0.751 (C) RMGIC + GLUMA: 3.304 ± 0.762 (D) RMGIC + AdheSE: 4.042 ± 0.742	Tensile strength RMGIC + AdheSE > RMGIC + GLUMA > RMGIC > RMGIC + Varnish	-	Recommends use of resin-based and glutaraldehyde-based sealers with RMGIC before crown cementation.
Lawaf et al., 2016, Iran [31]	In vitro	*n* = 20 (10 per group)	Human premolars	Finish line: Deep chamfer Axial height: 4 mm Taper: 6° Spacer: 3 coats Ageing: No	Full base metal alloy copings Fabrication technique: lost wax casting	No DA applied	Application of DA before final cementation	GLUMA (Heraeus-Kulzer, Hanau, Germany )	5% Glutaraldehyde & HEMA	Self-adhesive RC (RelyX U200, 3M ESPE, St. Paul, MN, USA)	TBS; UTM	Tensile Bond Strength (N) (A) RC: 164.45 ± 39.3 (B) RC + GLUMA DA: 230.63 ± 63.8	TBS RC + GLUMA DA > RC	-	Application of GLUMA DA on Hypersensitive prepared teeth before final cementation using self-adhesive RC.
Pilo et al., 2016, Israel [10]	In vitro	*n* = 40 (10 per group)	Human Mandibular molars	Finish line: Chamfer Axial height: 5 mm Taper: 10° Spacer: 50 µ Ageing: 10,000 cycles	Zirconia crowns copings (Lava frame Y-TZP blocks, 3M ESPE, Seefeld, Germany) Fabrication technique: CAD/CAM milling	No DA applied	Application of DA before final cementation	Colgate Sensitive Pro-Relief Desensitizing Paste (Colgate -Palmolive Company, New York, NY, USA)	8% arginine and calcium carbonate	(i) RMGIC (RelyX Luting 2, 3M ESPE) (ii) Self Adhesive RC (RelyX U-200, 3M ESPE)	Retentive strength, UTM	Retentive strength (MPa) (A) RMGIC + DA: 2.92 ± 0.84 (B) RMGIC: 3.16 ± 0.73 (C) Self Adhesive RC + DA: 2.27 ± 0.64 (D) Self Adhesive RC: 2.29 ± 0.55	Retentive strength RMGIC > RMGIC + DA > RC > RC + DA	Retentive strength RMGIC > RC	Retentive strengths of zirconia crowns cemented by either RMGIC or RC remain unaltered when 8% A-C-C is used as DA.
Mapkar et al., 2018, India [11]	In vitro	*n* = 33 (11 per group)	Human maxillary first premolars	Finish line: shoulder Axial height: 4 mm Taper: 20° Spacer: 1 layer Ageing: 2500 cycles	Full metal base metal alloy copings Fabrication technique: lost wax casting	No DA applied	Application of DA before final cementation	(A) Gluma (Heraeus Kulzer, hanau, Germany) (B) Ultraseal (Ultradent, South Jordan, UT USA)	(A) 5% Glutaraldehyde & HEMA (B) Non polymerizable, high -molecular-weight resin	ZPC (MEDIcept, Middlesex, UK).	Dislodgement force, UTM	Dislodgement force (N): (A) ZPC:345.01 (B) ZPC + Gluma:556.41 ZPC + Ultraseal: 320.22	Dislodgement force: ZPC + Gluma > ZPC > ZPC + Ultraseal	-	Significant increase in retention after application of Gluma DA, whereas non-significant decrease after Ultraseal application.
Pilo et al., 2018, Israel [39]	In vitro	*n* = 40 (10 per group)	Human Mandibular molars	Finish line: Chamfer Axial height: 5 mm Taper: 10° Spacer: 50 µ Ageing: 5000 cycles	Full metal Co-Cr alloy Fabrication technique: selective laser melting (SLM) technology	No DA applied	Application of DA before final cementation	Colgate Sensitive Pro-Relief Desensitizing Paste (Colgate-Palmolive Company, New York, NY, USA)	8% arginine and calcium carbonate	(i) GIC (ii) ZPC	Retentive strength, UTM	Retentive strength (MPa) GIC + DA: 6.39 ± 1.06 GIC: 5.73 ± 1.10 ZPC + DA: 2.39 ± 0.99 ZPC: 3.10 ± 1.44	Retentive strength: GIC + DA > GIC > ZPC > ZPC + DA	Retentive strength: GIC > ZPC	Application of 8% arginine and calcium carbonate can be used safely without reducing the retentive strength of crowns cemented with GIC and/or ZPC.
Asadullah et al., 2018, India [46]	In vitro	*n* = 33 (11 per group)	Human maxillary first premolars	Finish line: shoulder Axial height: 4 mm Taper: 20° Spacer: 1coat Ageing: 2500 cycles	Full base metal alloy copings Fabrication technique: lost wax casting	No DA applied	Application of DA before final cementation	(A) ULTRASEAL (Ultradent, South Jordan, UT, USA) (B) GLUMA (Heraeus-Kulzer, Hanau, Germany)	(A) non polymerizable, high -molecular-weight resin (B) 5% Glutaraldehyde & HEMA	RC (RelyX, 3M ESPE)	Dislodgement force, UTM	Dislodgement force (N) (A) RC: 228.892 ^##^ (B) RC + Ultra seal DA: 173.353 ^##^ (C) RC + GLUMA DA: 339.098 ^##^	Dislodgement force: RC + GLUMA > RC > RC + Ultra seal	-	GLUMA DA can be safely used with RC whereas, Ultraseal DA should not be used with RC.
Himashilpa et al., 2019, India [35]	In vitro	*n* = 420 (10 per group)	Human maxillary premolars	Finish line: Shoulder Axial height: 4 mm Taper: 12° Spacer: NM Ageing: No	Full metal nickel chromium alloy copings Fabrication technique: lost wax casting	No DA applied	Application of DA before final cementation	(A) Systemp (ivoclar vivadent, Liechtenstein) (B) Gluma (Heraeus Kulzer, Hanau, Germany) (C) GC tooth Mousse (GC International, Itabashiku, Tokyo, Japan) (D) Colgate Sensitive Pro-Relief Desensitizing Paste (Colgate-Palmolive Company, New York, NY, USA) (E) Sensodyne repair and protect (F) Sensodyne rapid action repair and protect	(A) Poly(ethylene glycol)dimethacrylate and glutaraldehyde (B) 5% Glutaraldehyde & HEMA (C) CPP-ACP (D) 8% arginine and calcium carbonate (E) Novamin (F) Fluoride	(A) GIC (Fuji luting GC, GC Corporation, Tokyo, Japan) (B) RMGIC: (RelyX Luting Cement 3M ESPE) (C) self-adhesive RC (Maxcem Elite, Kerr, Orange County, CA, USA)	TBS, UTM	TBS (N) Thermocycling (A) GIC: 6.79 ± 0.74 (B) GIC + Systemp: 7.75 ± 0.67 (C) GIC + Gluma: 6.89 ± 0.66 (D) GIC + Mousse: 6.88 ± 0.65 (E) GIC + Arginine: 6.40 ± 0.86 (F) GIC + Novamin: 6.39 ± 0.36 (G) GIC + Flouride: 6.59 ± 1.32 (H) RMGIC: 8.26 ± 0.64 (I) RMGIC + Systemp: 8.44 ± 0.51 (J) RMGIC + Gluma: 8.13 ± 0.49 (K) RMGIC + Mousse: 7.80 ± 0.59 (L) RMGIC + Arginine: 8.15 ± 0.96 (M) RMGIC + Novamin: 8.05 ± 0.42 (N) RMGIC + Flouride: 7.37 ± 1.10 (O) RC: 9.85 ± 0.85 (P) RC + Systemp: 10.80 ± 0.91 (Q) RC + Gluma: 10.06 ± 0.77 (R) RC + Mousse: 9.97 ± 0.82 (S) RC + Arginine: 9.63 ± 0.80 (T) RC + Novamin: 9.49 ± 0.87 (U) RC + Flouride: 9.17 ± 0.64 Non-Thermocycling (A) GIC: 5.41 ± 1.02 (B) GIC + Systemp: 6.15 ± 0.49 (C) GIC + Gluma: 5.61 ± 0.89 (D) GIC + Mousse: 6.85 ± 0.71 (E) GIC + Arginine: 6.29 ± 0.43 (F) GIC + Novamin: 5.86 ± 0.49 (G) GIC + Flouride: 6.15 ± 1.10 (H) RMGIC: 6.58 ± 1.32 (I) RMGIC + Systemp: 7.54 ± 0.77 (J) RMGIC + Gluma: 7.47 ± 0.98 (K) RMGIC + Mousse: 7.35 ± 1.10 (L) RMGIC + Arginine: 6.54 ± 0.89 (M) RMGIC + Novamin:7.54 ± 0.34 (N) RMGIC + Flouride: 6.97 ± 0.61 (O) RC: 9.17 ± 0.52 (P) RC + Systemp: 9.25 ± 0.78 (Q) RC + Gluma: 9.12 ± 0.59 (R) RC + Mousse: 8.80 ± 0.78 (S) RC + Arginine: 8.64 ± 0.60 (T) RC + Novamin:8.75 ± 0.58 (U) RC + Flouride: 8.74 ± 0.64	TBS: Thermocycling Resin Cement: RC + Systemp > RC + Gluma > RC + Mousse > RC > RC + Arginine > RC + Novamin > RC + Flouride RMGIC: RMGIC + Systemp > RMGIC > RMGIC + Arginine > RMGIC + Gluma > RMGIC + Novamin > RMGIC + Mousse > RMGIC + Flouride GIC: GIC + Systemp > GIC + Gluma > GIC + Mousse > GIC > GIC + Flouride > GIC + Arginine > GIC + Novamin	TBS: RC > RMGIC > GIC	Highest TBS displayed by use of systemp DA, and lowest by Pro-Arginine in all groups. Thermocycling increased TBS
Supraja et al., 2020, India [47]	In vitro	*n* = 45 (5 per group)	Human Maxillary premolars	Finish line: Chamfer Axial height: 4 mm Taper: 6° Spacer: NM Ageing: No	Full metal Co-Cr alloy Fabrication technique: additive manufacturing (direct metal laser sintering).	No DA applied	Application of DA before final cementation	(A) A-CC-F DA (custom made) (B) CPP-ACP-F DA (custom made)	(A) Arginine, Calcium Carbonate, Fluoride (B) Casein Phosphopeptide, Amorphous Calcium Phosphate, Fluoride	(i) GIC (NM) (ii) RMGIC (NM) (iii) RC (NM)	TBS; UTM	TBS (N): GIC + A-CC-F DA: 90.26 ± 10.68 GIC + CPP-ACP-F DA: 272.32 ± 30.5 GIC: 308.62 ± 58.84 RMGIC + A-CC-F DA: 85.07 ± 18.82 RMGIC + CPP-ACP-F DA: 203.47 ± 60.57 RMGIC: 176.89 ± 35.46 RC + A-CC-F DA: 236.05 ± 43.62 RC + CPP-ACP-F DA: 158.66 ± 25.32 RC+: 300.35 ± 27.9	TBS: GIC: GIC > GIC + A-CC-F DA > GIC + CPP-ACP-F DA RMGIC: RMGIC + CPP-ACP-F DA > RMGIC > RMGIC + A-CC-F DA RC: RC > RC + A-CC-F DA > RC + CPP-ACP-F DA	TBS: RC > RMGIC > GIC	Application of both types of DA decreased TBS for GIC to dentin Application of CPP-ACP-F DA increased, while A-CC-F DA decreased the TBS for RMGIC to dentin Application of both types of DA decreased TBS for RC to dentin
Hanjik et al., 2021, Syria [48]	In vitro	*n* = 40 (10 per group)	Human Maxillary premolars	Finish line: Chamfer Axial height: 4 mm Taper: 6° Spacer: 2 layer, 1 mm above the finish line. Ageing: No	Full metal Ni-Cr crown Fabrication technique: lost wax casting	No DA applied	Application of DA before final cementation	Systemp desensitizer (ivoclar vivadent, Schaan, Liechtenstein)	Poly(ethylene glycol)dimethacrylate and glutaraldehyde in an aqueous solution	(i) GIC (Cavex, CJ Haarlem, The Netherlands) (ii) RMGIC (GC Fuji plus, Tokyo Japan)	TBS; UTM	TBS (N): RMGIC + DA: 829.95 ±104.29 RMGIC + No DA:604.03 ± 127.20 GIC + DA: 415.74 ± 139.92 GIC + No DA: 433.74 ± 177.73	TBS: DA + RMGIC > RMGIC > GIC > DA + GIC	TBS: RMGIC > GIC	Application of DA increase TBS for RMGIC to dentin Application of DA decrease TBS for GIC to dentin
Dewan et al., 2022; Saudi Arabia [49]	In vitro	*n* = 40 (10 per group)	Human molars	Finish line: Chamfer Axial height: 4 mm Taper: 10° Spacer: NM Ageing: 3000 cycles	Zirconia copings (Ceramill ZI, Austria) Fabrication technique: CAD/CAM milling	No DA applied	Application of DA before final cementation	(A) Gluma (Heraeus Kulzer, Hanau, Germany) (B) Telio CS (Ivoclar Vivadent, Schaan, Liechtenstein) (C) Shield Force Plus (Tokuyama Dental, Encinitas, CA, USA)	(A) 5% Glutaraldehyde & HEMA (B) PEGDMA, Glutaraldehyde (C) HPDMA & PA	RC (Rely X U200, 3M ESPE, St. Paul, MN, USA )	TS, UTM	TS (MPa) (A) RC: 0.22 ± 0.03 (B) RC + Gluma: 0.53 ± 0.08 (C) RC + Telio CS: 0.35 ± 0.10 (D) RC + Shield force: 0.36 ± 0.14	TS: RC + Gluma > RC + Shield force > Rc + Telio CS > RC	-	Advocates using the tested DAs before cementing Zirconia crowns.

TBS: tensile bond strength; DA: desensitizing agent; RMGIC: resin-modified glass ionomer cement; Ni-Cr: nickel chromium; Co-Cr: cobalt chromium; A-C-C-F: arginine–calcium carbonate–fluoride; A-C-C: arginine–calcium carbonate; CPP-ACP-F: casein phosphopeptide–amorphous calcium phosphate–fluoride; NM: not mentioned, RC: resin cement; ZPC: zinc phosphate cement; UTM: universal testing machine; Er, Cr: YSGG: erbium, chromium:yttrium, selenium, galium, garnet; NM: not mentioned; GLU: glutaraldehyde; D-TMR: di- and trimethacrylate resin; SP: seal and protect; TM: tooth MousseMousse; PENTA: dipentaerythritol penta acrylate monophosphate; HEMA: 2-hydroxyethyl-methacrylate; PCC: polycarboxylate cement; NTG-GMA: N-olyglycine glycidyl methacrylate; BPDM: biphenyl dimethacrylate; UDMA: urethane dimethacrylate; TEGMA: tolnyl ethyl glycidal dimethacrylate; PEGDMA: polyethylene glycol dimethacrylate; HPDMA: hydroxy propoxy dimethacrylate; PA: phosphoric acid; ^##^: data retrieved from plot digitizer app.

**Table 2:** Quality analyses of the included studies using the modified CONSORT scale.

Item →	1	2a	2b	3	4	5	6	7	8	9	10	11	12	13	14
Studies															
Mausner et al., 1996 [41]	Y	Y	Y	Y	Y	N	Y	N	N	N	Y	Y	N	N	N
Swift et al., 1997 [36]	Y	Y	Y	Y	Y	N	Y	N	N	N	Y	Y	N	N	N
Johnson et al., 1998 [34]	Y	Y	Y	Y	Y	N	N	N	N	N	Y	Y	N	N	N
Yim et al., 2000 [37]	Y	Y	Y	Y	Y	N	Y	N	N	N	Y	Y	Y	Y	N
Wolfart et al., 2003 [12]	Y	Y	Y	Y	Y	N	Y	N	N	N	Y	Y	Y	N	N
Johnson et al., 2004 [42]	Y	Y	Y	Y	Y	N	N	N	N	N	Y	Y	N	N	N
Sipahi et al., 2007 [38]	Y	Y	Y	Y	Y	N	N	N	N	N	Y	Y	N	N	N
Jalandar et al., 2012 [18]	Y	Y	Y	Y	Y	N	N	N	N	N	Y	Y	Y	N	N
Stawarczyk et al., 2012 [19]	Y	Y	Y	Y	Y	N	Y	N	N	N	Y	Y	Y	N	N
Patel et al., 2013 [20]	Y	Y	Y	Y	Y	N	N	N	N	N	Y	Y	Y	N	N
Chandrasekaran et al., 2014 [43]	Y	Y	Y	Y	Y	N	Y	Y	N	N	Y	Y	Y	N	N
Kumar et al., 2015 [44]	Y	Y	Y	Y	Y	N	N	N	N	N	Y	Y	N	N	N
Chandavarkar et al., 2015 [8]	Y	Y	Y	Y	Y	N	N	N	N	N	Y	Y	Y	Y	N
Janapala et al., 2015 [45]	Y	Y	Y	Y	Y	Y	N	N	N	N	Y	Y	Y	Y	N
Lawaf et al., 2016 [31]	Y	Y	Y	Y	Y	N	N	N	N	N	Y	Y	Y	N	N
Pilo et al., 2016 [10]	Y	Y	Y	Y	Y	N	N	N	N	N	Y	Y	N	N	N
Mapkar et al., 2018 [11]	Y	Y	Y	Y	Y	N	N	N	N	N	Y	Y	Y	Y	N
Pilo et al., 2018 [39]	Y	Y	Y	Y	Y	N	N	N	N	N	Y	Y	Y	Y	N
Asadullah et al., 2018 [46]	Y	Y	Y	Y	Y	N	N	N	N	N	Y	Y	N	Y	N
Himashilpa et al., 2019 [35]	Y	Y	Y	Y	Y	N	N	N	N	N	Y	Y	N	N	N
Supraja et al., 2020 [47]	Y	Y	Y	Y	Y	Y	N	N	N	N	Y	Y	Y	Y	N
Hanjik et al., 2021 [48]	Y	Y	Y	Y	Y	N	N	N	N	N	Y	Y	N	N	N
Dewan et al., 2022 [49]	Y	Y	Y	Y	Y	N	N	N	N	N	Y	Y	Y	Y	Y

## Data Availability

The data that support the findings of this study are available from the corresponding author upon reasonable request.

## Supplementary Materials

The following supporting information can be downloaded at: https://www.mdpi.com/article/10.3390/medicina59030515/s1, Table S1: Search strategy for the electronic databases.
