# Supplementary material for: The Effect of Dentine Desensitizing Agents on the Retention of Cemented Fixed Dental Prostheses: A Systematic Review
Source: Medicina (Kaunas). 2023 Mar 6;59(3):515. doi: 10.3390/medicina59030515 (PMC10051248; doi:10.3390/medicina59030515)
Supplement: Supplementary file 1 [file medicina-59-00515-s001.zip › medicina-2235376-supplementary.pdf]

Supplementary Table S1. Search strategy for the electronic databases.

Electronic database	Search strategy and search terms	Number of Titles
MEDLINE-PUBMED	(("dental restoration, permanent"[MeSH Terms] OR "Dental Prosthesis"[MeSH Terms] OR crowns[MeSH Terms] OR "denture, partial, fixed"[MeSH Terms] OR "Dental cements"[MeSH Terms] OR "Resin cements"[MeSH Terms] OR "Complete cast crowns"[All Fields] OR "luting agent*"[All Fields] OR "Self-adhesive resin cement"[All Fields] OR "luting cement*"[All Fields] OR "Adhesive cementation"[All Fields] OR "glass ionomer cement"[All Fields] OR "adhesive resin cement"[All Fields] OR "Fixed dental prosthesis"[All Fields]) AND ("Dentin Desensitizing Agents"[MeSH Terms] OR "Dentine Desensitizing"[All Fields] OR "Desensitizing agents"[All Fields] OR "Dentin pretreatment"[All Fields] OR "Dentin desensitizer"[All Fields] OR "Desensitizing paste"[All Fields] OR (gluma[Supplementary Concept] OR gluma[All Fields] OR gluma[All Fields]) OR Systemp[All Fields]) AND ("Dental Prosthesis Retention"[MeSH Terms] OR "Prosthesis Retention"[MeSH Terms] OR "bond strength"[All Fields] OR Microtensile[All Fields] OR "tensile bond strength"[All Fields] OR "dentine bond strength"[All Fields])) AND ((humans[Filter]) AND (english[Filter]))	176
	#1 MeSH descriptor: [Dental Restoration, Permanent] explode all trees<br>#2 MeSH descriptor: [Dental Prosthesis] explode all trees<br>#3 MeSH descriptor: [Crowns] explode all trees<br>#4 MeSH descriptor: [Denture, Partial, Fixed] explode all trees<br>#5 MeSH descriptor: [Dental Cements] explode all trees<br>#6 MeSH descriptor: [Resin Cements] explode all trees<br>#7 “Complete cast crowns”<br>#8 “Luting agent*”<br>#9 “Self-adhesive resin cement”<br>#10 “luting cement*”<br>#11 “Adhesive cementation”<br>#12 “glass ionomer cement”<br>#13 “adhesive resin cement”	

Cochrane Library	#14 “Fixed dental prosthesis”<br>#15 MeSH descriptor: [Dentin Desensitizing Agents] explode all trees<br>#16 “Dentine Desensitizing”<br>#17 “Desensitizing agents”<br>#18 “Dentin pretreatment”<br>#19 “Dentin desensitizer”<br>#20 “Desensitizing paste”<br>#21 Gluma<br>#22 Systemp<br>#23 MeSH descriptor: [Dental Prosthesis Retention] explode all trees<br>#24 MeSH descriptor: [Prosthesis Retention] explode all trees<br>#25 “bond strength”<br>#26 Microtensile<br>#27 “tensile bond strength”<br>#28 “dentine bond strength”<br>#29 ("microtensile bond strength"):ti,ab,kw<br>#33<br>#1 OR #2 OR #3 OR #4 OR #5 OR #6 #7 OR #8 OR #9<br>OR #10 OR #11 OR #12 OR #13 OR #14<br>#34<br>#15 OR #16 OR #17 #18 OR #19 OR #20 #21 OR #22<br>#35<br>#23 OR #24 OR #25 OR #26 OR #27 OR #28 #29<br>#36 #33 AND #34 AND #35	5
Scopus	(("dental restoration, permanent" OR "Dental Prosthesis" OR crowns OR "denture, partial, fixed" OR "Dental cements" OR "Resin cements" OR "Complete cast crowns" OR "luting agent*" OR "Self-adhesive resin cement" OR "luting cement*" OR "Adhesive cementation" OR "glass ionomer cement" OR "adhesive resin cement" OR "Fixed dental prosthesis") AND ("Dentin Desensitizing Agents" OR "Dentine Desensitizing" OR "Desensitizing agents" OR "Dentin pretreatment" OR "Dentin desensitizer" OR "Desensitizing paste" OR gluma OR systemp) AND ("Dental Prosthesis Retention" OR "Prosthesis Retention" OR "bond strength" OR microtensile OR "tensile bond	1206

	strength" OR "dentine bond strength")) AND (LIMIT-TO (PUBSTAGE , "final") OR LIMIT-TO (PUBSTAGE , "aip")) AND (LIMIT-TO (DOCTYPE , "ar") OR LIMIT-TO (DOCTYPE , "re")) AND (LIMIT-TO (SUBJAREA , "DENT") OR LIMIT-TO (SUBJAREA , "MATE") OR LIMIT-TO (SUBJAREA , "HEAL")) AND (LIMIT-TO (LANGUAGE , "English"))	
Web of Science (Core Collection)	((ALL=(("dental restoration, permanent" OR "Dental Prosthesis" OR crowns OR "denture, partial, fixed" OR "Dental cements" OR "Resin cements" OR "Complete cast crowns" OR "luting agent*" OR "Self-adhesive resin cement" OR "luting cement*" OR "Adhesive cementation" OR "glass ionomer cement" OR "adhesive resin cement" OR "Fixed dental prosthesis"))) AND ALL=(("Dentin Desensitizing Agents" OR "Dentine Desensitizing" OR "Desensitizing agents" OR "Dentin pretreatment" OR "Dentin desensitizer" OR "Desensitizing paste" OR gluma OR systemp))) AND ALL=(("Dental Prosthesis Retention" OR "Prosthesis Retention" OR "bond strength" OR microtensile OR "tensile bond strength" OR "dentine bond strength"))	67
